# Risk factors for in-hospital mortality and secondary bacterial pneumonia among hospitalized adult patients with community-acquired influenza: a large retrospective cohort study

**DOI:** 10.1186/s13756-023-01234-y

**Published:** 2023-03-31

**Authors:** Guangzhao Yi, Marlieke E. A. de Kraker, Niccolò Buetti, Xiaoni Zhong, Jinyan Li, Zhe Yuan, Weimin Zhu, Jia Zhou, Hongyu Zhou

**Affiliations:** 1 Department of Hospital Infection Control, The First Affiliated Hospital of Chongqing Medical University, You Yi Road 1, Chongqing, 400016 China; 2 Infection Control Program, Faculty of Medicine, Geneva University Hospitals, Geneva, Switzerland; 3 Research Center for Medicine and Social Development, School of Public Health, Chongqing Medical University, Chongqing, China; 4 Information Center, The First Affiliated Hospital of Chongqing Medical University, Chongqing, China; 5 Department of Infectious Diseases, The First Affiliated Hospital of Chongqing Medical University, Chongqing, China; 6 Department of Pulmonary and Critical Care Medicine, The First Affiliated Hospital of Chongqing Medical University, Chongqing, China; 7 Department of Disease Prevention and Health Protection, The First Affiliated Hospital of Chongqing Medical University, Chongqing, China

**Keywords:** Risk factors, Seasonal influenza, Secondary bacterial pneumonia, All-cause in-hospital mortality

## Abstract

**Background:**

Secondary bacterial pneumonia is an important complication of seasonal influenza, but little data is available about impact on death and risk factors. This study identified risk factors for all-cause in-hospital mortality and secondary bacterial pneumonia among hospitalized adult patients with community-acquired influenza.

**Methods:**

A retrospective cohort study was performed at a tertiary teaching hospital in southwest China. The study cohort included all adult hospitalized patients with a laboratory-confirmed, community-acquired influenza virus infection during three consecutive influenza seasons from 2017 to 2020. Cause-specific Cox regression was used to analyze risk factors for mortality and secondary bacterial pneumonia, respectively, accounting for competing events (discharge alive and discharge alive or death without secondary bacterial pneumonia, respectively).

**Results:**

Among 174 patients enrolled in this study, 14.4% developed secondary bacterial pneumonia and 11.5% died during hospitalization. For all-cause in-hospital mortality, time-varying secondary bacterial pneumonia was a direct risk factor of death (cause-specific hazard ratio [csHR] 3.38, 95% confidence interval [CI] 1.25–9.17); underlying disease indirectly increased death risk through decreasing the hazard of being discharged alive (csHR 0.55, 95% CI 0.39–0.77). For secondary bacterial pneumonia, the final model only confirmed direct risk factors: age ≥ 65 years (csHR 2.90, 95% CI 1.27–6.62), male gender (csHR 3.78, 95% CI 1.12–12.84) and mechanical ventilation on admission (csHR 2.96, 95% CI 1.32–6.64).

**Conclusions:**

Secondary bacterial pneumonia was a major risk factor for in-hospital mortality among adult hospitalized patients with community-acquired influenza. Prevention strategies for secondary bacterial pneumonia should target elderly male patients and critically ill patients under mechanical ventilation.

**Supplementary Information:**

The online version contains supplementary material available at 10.1186/s13756-023-01234-y.

## Background

Seasonal influenza has an important impact on public health. During epidemics, attack rates of influenza in unvaccinated populations were estimated to be 10–20% globally [1]. Most people recover from mild self-limiting illness, but more frail people can develop severe illness increasing their risk of mortality [2]. Annually, influenza epidemics result in about three million to five million cases of severe illness and about 290,000 to 650,000 associated deaths worldwide, making it one of the major public health problems [2].

One of the more important epidemics, the 2009 influenza A (H1N1) pandemic, was estimated to have caused between 150,000 and 575,000 excess deaths, increasing the concern about the possible adverse outcomes of influenza and an interest in underlying risk factors [3]. However, the risk factors of adverse outcomes caused by seasonal influenza after the 2009 pandemic, received much less attention. Moreover, few studies in this field included data on Chinese populations, especially those in southwest China.

Pneumonia is one of the most common complications of influenza [4]. It can be caused by primary virus infection, and/or secondary bacterial infection which can occur during or following initial infection from influenza virus [5, 6]. Secondary bacterial infection can increase the risk of death in patients with viral respiratory infectious disease including influenza, but it is an underestimated problem [5–8]. Despite clinical similarities, secondary bacterial pneumonia differs from primary viral pneumonia caused by influenza in pathogen, timing of onset, risk factors, and even outcomes [9, 10], so it may require different prevention and control measures. However, most studies on influenza patients focus on the risk factors for viral pneumonia and its impact on death, rather than those for secondary bacterial pneumonia [11, 12]. The effect of secondary bacterial pneumonia on death is often disregarded, and its risk factors are not well understood.

Thus, based on the hypothesis that secondary bacterial pneumonia is an important risk factor for death among patients with influenza, and further focusing on risk factors for secondary bacterial pneumonia, a retrospective cohort study of hospitalized adult patients with community-acquired influenza in a large tertiary teaching hospital in China was performed to identify the risk factors for all-cause in-hospital mortality and secondary bacterial pneumonia.

## Methods

### Study design

A retrospective cohort study was carried out at a tertiary comprehensive, teaching hospital in Chongqing, China for three consecutive influenza seasons (2017/2018, 2018/2019 and 2019/2020). The hospital, with 3200 authorized beds, served about 8,580,775 outpatients and 369,955 in-patients in 2017, 2018 and 2019. It has been one of the sentinel hospitals for influenza surveillance in southwest China since 2005. The Research Ethics Board of the hospital approved this study and granted waiver of patient informed consent considering this is not an intervention study (approval number: 2021–356).

### Study population

The study cohort included all adult patients with a confirmed, community-acquired influenza virus infection during one of the three included influenza seasons (from November of 2017, 2018 and 2019 to March of the following year, later referred to as INF2017, INF2018 and INF2019). The inclusion criteria included: (1) age ≥ 15 years; (2) hospitalized for at least 24 h; (3) confirmed influenza cases which met a laboratory criterion (positive influenza virus nucleic acid testing) and at least one clinical criterion (fever, headache, muscle or body aches, cough, sore throat, runny or stuffy nose, or systemic symptoms such as chills and fatigue, and infrequent digestive symptoms such as vomiting and diarrhea, or atypical symptoms probably due to an infection based on clinicians’ judgment) according to the Diagnosis and Treatment Scheme for Influenza (2020 revised edition) released by the National Health Commission of the People’s Republic China [13]; (4) community-acquired influenza acquisition (*i.e.*, symptom onset of confirmed influenza cases before admission or within 7 days (the longest incubation period of influenza [13, 14]) of admission [15, 16]). All included patients in the cohort were followed from admission to discharge or death in the hospital.

### Data collection

An experienced clinician pre-reviewed electronic medical records of included cases from the hospital clinical information system with an infection control professional, to determine study variables. Collected variables included demographics, epidemiology, hospitalization information, underlying disease, self-reported symptoms on admission, the laboratory findings on admission, the first radiographic findings after admission, treatments, secondary bacterial pneumonia including microbiological characteristics of the causative pathogen(s), and all-cause in-hospital mortality (Additional file 1). An information engineer from the medical information department of the hospital extracted data, and an experienced clinician checked data consistency and completeness through reviewing electronic medical records. Vaccine status data was derived from the Chongqing Immunization Information System which records every vaccination given to Chinese citizens.

### Definitions

Secondary bacterial pneumonia was defined as a pulmonary bacterial/fungal infection that occurred after an influenza virus infection, diagnosed by a recurrence of symptoms or a demonstrable new pulmonary infiltrate by chest radiograph or other imaging technique plus one or more positive bacterial/fungal cultures obtained from blood, pleural fluid, endotracheal aspirate, bronchoalveolar lavage or sputum ≥ 48 h after admission [5, 11, 17]. Secondary bacterial pneumonia included ventilator-associated pneumonia (VAP). VAP was defined as pneumonia developed in patients who were under invasive mechanical ventilation (MV, endotracheal intubation or tracheotomy) for at least 48 h [18]. Microbiological diagnosis was considered “definite” only when the organism was isolated from blood or pleural fluid, while it was considered “probable” when the organism was isolated from qualified sputum (sample with > 25 leukocytes and < 10 epithelial cells per × 100 magnification field), endotracheal aspirate or bronchoalveolar lavage fluid [19–22]. All-cause in-hospital mortality was defined as death from any cause during hospitalization.

### Microbiologic analyses

If there was a clinical suspicion of community-acquired influenza, routine nasopharyngeal swabs were collected from patients, if clinically indicated, lower respiratory tract specimens (sputum, bronchoalveolar lavage fluid or tracheal aspirate) were collected as well. Real-time reverse transcriptase polymerase chain reaction (RT-PCR) assay for influenza virus nucleic acid testing was used to confirm the influenza virus infection. All RT-PCR assays were conducted in the Infection Laboratory of the hospital or the Virology Laboratory of the local Centers for Disease Control and Prevention (CDC), which were qualified to test for influenza virus, and the results were typically available within 24 h. Total 6654 samples were tested for influenza virus infection by RT-PCR assays during INF2017, INF2018 and INF2019.

If a bacterial/fungal infection was suspected by clinicians during the patient’s hospitalization, routine blood culture and sputum culture were performed immediately in the Microbiology Laboratory of the hospital. If clinically indicated, pleural fluid, tracheal aspirate or bronchoalveolar lavage fluid were also sent for microbiological evaluation. The Microbiology Laboratory identified the isolates and performed routine antimicrobial susceptibility testing according to standard microbiological procedures.

### Statistical analyses

Continuous variables were described as median and interquartile range (IQR), and categorical variables were presented as counts (percentage). The Mann–Whitney *U* test was used for comparing continuous variables. The chi-squared test or Fisher’s exact test was used to compare categorical variables. For the variables with a low number of missing values, patients with missing data were excluded when comparing these variables.

Cox regression analysis was performed to identify risk factors for all-cause in-hospital mortality and secondary bacterial pneumonia. The day of admission was considered to be day 0 for the cohort. For the first analysis, focusing on risk factors for mortality, date of death was the endpoint, and secondary bacterial pneumonia was included as a time-varying covariable; for risk factor analysis for secondary bacterial pneumonia the day of first culture that later confirmed the pneumonia was the endpoint. For continuous variables, plotting the martingale residuals was used to check linearity. In case of nonlinearity, continuous variables were categorized, and cut-offs estimated based on the lowest log-rank p-value. All variables with p values < 0.20 in the univariable analysis were considered for the multivariable regression analysis. Collinearity was checked by the variance inflation factor, if needed the clinically most relevant variable was selected. In order to take into account competing risks, the cause-specific proportional hazards model was applied. For in-hospital mortality, discharge alive was considered a competing risk event; for secondary bacterial pneumonia, discharge alive or death without secondary bacterial pneumonia was considered as competing risk events. The proportional hazard (PH) assumption was tested by Schoenfeld residuals. The final model was selected using the best subset method based on Akaike’s Information Criteria (AIC) values. In the final models, a p-value < 0.05 was considered statistically significant. The Nelson-Aalen estimator was used for the cumulative hazard function and constructing the curve. The analyses were performed using R software, version 4.1.0.

## Results

Overall, 199 hospitalized patients with confirmed influenza, aged ≥ 15 years, were identified in the hospital during INF2017, INF2018 and INF2019. Of these, 198 patients were hospitalized for at least 24 h, and a total of 174 patients acquired influenza in the community and could be included in this study.

### Demographic and clinical characteristics of the study cohort

The demographic and clinical characteristics of the study cohort are shown in Table 1. Most cases (108/174 [62.1%]) were identified in the 2018/2019 influenza season. 157/174 (90.2%) and 15/174 (8.6%) patients were respectively infected with influenza A and B virus, and only two (1.1%) patients were infected with both viruses. The median age was 61.5 years (IQR, 44.0–71.0). Males accounted for 60.9% (106/174). Of all patients, 70.7% (123/174) had at least one underlying disease. Hypertension (48/174 [27.6%]), diabetes (38/174 [21.8%]) and chronic heart disease (37/174 [21.3%]) were the most common underlying diseases. A minority of patients, 30.5% (53/174), were current smokers. None of the patients received influenza vaccine > 14 days before symptom onset in the same season, and only one (0.6%) patient received pneumococcal vaccine in the last five years. None of the patients admitted since February 2020 was co-infected with COVID-19, all of these patients tested negative for SARS-CoV-2.

**Table 1:** Characteristics of hospitalized adult patients with community-acquired influenza included in this study

Variables	Total patients (N = 174)	All-cause in-hospital mortality			Secondary bacterial pneumonia		
		Died patients (n = 20)	Survived patients (n = 154)	p value	Patients with secondary bacterial pneumonia (n = 25)	Patients without secondary bacterial pneumonia (n = 149)	p value
*Influenza season*, n (%)							
2017	36 (20.7)	8 (40.0)	28 (18.2)	**0.049**	5 (20.0)	31 (20.8)	0.975
2018	108 (62.1)	9 (45.0)	99 (64.3)		16 (64.0)	92 (61.7)	
2019	30 (17.2)	3 (15.0)	27 (17.5)		4 (16.0)	26 (17.4)	
*Type of influenza virus (A or/and B)*, n (%)							
Type A	157 (90.2)	19 (95.0)	138 (89.6)	0.716	23 (92.0)	134 (89.9)	1.000
Type B	15 (8.6)	1 (5.0)	14 (9.1)		1 (4.0)	14 (9.4)	
Type A and Type B	2 (1.1)	0 (0.0)	2 (1.3)		1 (4.0)	1 (0.7)	
Age, median (IQR), years	61.5 (44.0–71.0)	65.0 (52.8–71.0)	60.0 (43.0–71.0)	0.176	68.0 (53.0–72.0)	59.0 (43.0–71.0)	0.101
Age ≥ 65 years, n (%)	72 (41.4)	12 (60.0)	60 (39.0)	0.072	16 (64.0)	56 (37.6)	**0.013**
Male gender, n (%)	106 (60.9)	16 (80.0)	90 (58.4)	0.063	22 (88.0)	84 (56.4)	**0.003**
Underlying disease, n (%)	123 (70.7)	18 (90.0)	105 (68.2)	**0.044**	21 (84.0)	102 (68.5)	0.114
Hypertension	48 (27.6)	9 (45.0)	39 (25.3)	0.064	11 (44.0)	37 (24.8)	**0.047**
Diabetes	38 (21.8)	6 (30.0)	32 (20.8)	0.515	8 (32.0)	30 (20.1)	0.184
Chronic pulmonary disease	30 (17.2)	2 (10.0)	28 (18.2)	0.551	7 (28.0)	23 (15.4)	0.210
COPD	28 (16.1)	2 (10.0)	26 (16.9)	0.642	6 (24.0)	22 (14.8)	0.385
Asthma	4 (2.3)	1 (5.0)	3 (1.9)	0.389	1 (4.0)	3 (2.0)	0.465
TB	3 (1.7)	0 (0.0)	3 (1.9)	1.000	1 (4.0)	2 (1.3)	0.374
Chronic heart disease^a^	37 (21.3)	5 (25.0)	32 (20.8)	0.886	4 (16.0)	33 (22.1)	0.487
Chronic renal disease	12 (6.9)	2 (10.0)	10 (6.5)	0.910	1 (4.0)	11 (7.4)	0.848
Chronic liver disease	29 (16.7)	4 (20.0)	25 (16.2)	0.915	3 (12.0)	26 (17.4)	0.699
Haematological disease	11 (6.3)	1 (5.0)	10 (6.5)	1.000	1 (4.0)	10 (6.7)	0.943
Cerebrovascular disease	10 (5.7)	0 (0.0)	10 (6.5)	0.507	2 (8.0)	8 (5.4)	0.953
Malignancy	18 (10.3)	2 (10.0)	16 (10.4)	1.000	4 (16.0)	14 (9.4)	0.517
Current smoker, n (%)	53 (30.5)	10 (50.0)	43 (27.9)	**0.044**	11 (44.0)	42 (28.2)	0.112
*Self-reported symptoms on admission*, n (%)							
Fever	144 (82.8)	17 (85.0)	127 (82.5)	1.000	20 (80.0)	124 (83.2)	0.914
Cough	159 (91.4)	20 (100.0)	139 (90.3)	0.300	23 (92.0)	136 (91.3)	1.000
Sore throat	26 (14.9)	0 (0.0)	26 (16.9)	0.097	2 (8.0)	24 (16.1)	0.454
Dyspnoea	38 (21.8)	7 (35.0)	31 (20.1)	0.220	7 (28.0)	31 (20.8)	0.420
Chest pain	9 (5.2)	1 (5.0)	8 (5.2)	1.000	2 (8.0)	7 (4.7)	0.840
Muscle or body aches	32 (18.4)	1 (5.0)	31 (20.1)	0.181	1 (4.0)	31 (20.8)	0.084
Fatigue	38 (21.8)	5 (25.0)	33 (21.4)	0.939	3 (12.0)	35 (23.5)	0.198
Headaches	16 (9.2)	0 (0.0)	16 (10.4)	0.271	1 (4.0)	15 (10.1)	0.550
Gastrointestinal symptoms	19 (10.9)	4 (20.0)	15 (9.7)	0.316	3 (12.0)	16 (10.7)	1.000
*The laboratory findings on admission*							
Neutropenia^b^, n (%)	2/173 (1.2)	0 (0.0)	2/153 (1.3)	1.000	0 (0.0)	2/148 (1.4)	1.000
Lymphocytopenia^c^, n (%)	32/172 (18.6)	9 (45.0)	23/152 (15.1)	**0.003**	5 (20.0)	27/147 (18.4)	1.000
Thrombocytopenia^d^, n (%)	37/173 (21.4)	9 (45.0)	28/153 (18.3)	**0.014**	6 (24.0)	31/148 (20.9)	0.731
Anaemia^e^, n (%)	52/173 (30.1)	12 (60.0)	40/153 (26.1)	**0.002**	13 (52.0)	39/148 (26.4)	**0.010**
Hypoalbuminemia^f^, n (%)	45/172 (26.2)	13 (65.0)	32/152 (21.1)	**< 0.001**	12 (48.0)	33/147 (22.4)	**0.007**
*The first radiographic findings after admission*, n (%)							
Pleural effusion	37/163 (22.7)	5/18 (27.8)	32/145 (22.1)	0.805	10/23 (43.5)	27/140 (19.3)	**0.010**
Diffuse bilateral pulmonary infiltration	22/163 (13.5)	7/18 (38.9)	15/145 (10.3)	**0.003**	3/23 (13.0)	19/140 (13.6)	1.000
Mechanical ventilation^g^ on admission, n (%)	35 (20.1)	15 (75.0)	20 (13.0)	**< 0.001**	11 (44.0)	24 (16.1)	**0.001**
Neuraminidase inhibitors treatment, n (%)	162 (93.1)	19 (95.0)	143 (92.9)	1.000	22 (88.0)	140 (94.0)	0.508
Time from symptom onset to neuraminidase inhibitors treatment, median (IQR), days	4.5/162 (2.0–8.0)	5.0/19 (2.0–7.0)	4.0/143 (2.0–8.0)	0.845	5.5/22 (2.0–8.0)	4.0/140 (2.0–8.0)	0.869
Neuraminidase inhibitors treatment ≤ 48 h since symptom onset	44 (25.3)	5 (25.0)	39 (25.3)	0.975	6 (24.0)	38 (25.5)	0.873
Duration of neuraminidase inhibitors treatment, median (IQR), days	5.9/162 (4.0–9.7)	8.0/19 (5.4–11.0)	5.8/143 (3.9–9.5)	0.141	6.4/22 (4.8–13.6)	5.9/140 (3.9–9.2)	0.113
Secondary bacterial pneumonia, n (%)	25 (14.4)	7 (35.0)	18 (11.7)	**0.014**	–	–	–
Time from symptom onset to secondary bacterial pneumonia, median (IQR), days	8.0/25 (4.5–15.0)	9.0/7 (3.0–19.0)	7.5/18 (4.8–14.5)	0.976	–	–	–
Length of hospital stay, median (IQR), days	11.0 (6.0–17.0)	10.0 (6.0–14.0)	11.0 (6.0–17.0)	0.394	14.0 (7.0–19.0)	11.0 (6.0–16.0)	0.131

*IQR* interquartile range; *COPD* chronic obstructive pulmonary disease; *TB* tuberculosis

Bold texts indicate p value < 0.05

^a^Chronic heart disease included coronary heart disease, congestive heart failure, rheumatic heart disease, hypertensive heart disease, cor pulmonale and congenital heart disease

^b^Neutropenia: neutrophil count < 1500/mm^3^

^c^Lymphocytopenia: lymphocyte count < 800/mm^3^

^d^Thrombocytopenia: platelet count < 100,000/mm^3^

^e^Anaemia: haemoglobin < 120 g/L for men and < 110 g/L for women

^f^Hypoalbuminemia: albumin < 35 g/L

^g^Invasive and non-invasive mechanical ventilation were included

Cough (159/174 [91.4%]) and fever (144/174 [82.8%]) were the most common symptoms on admission. The median time from symptom onset to hospitalization was 3.0 days (IQR, 0.0–7.0). 32/172 (18.6%), 37/173 (21.4%), 52/173 (30.1%) and 45/172 (26.2%) patients had lymphocytopenia, thrombocytopenia, anaemia and hypoalbuminemia on admission, respectively. Among 163 patients with a first chest radiographic examination close to their date of admission, 37 (22.7%) and 22 (13.5%) developed pleural effusion and diffuse bilateral pulmonary infiltration, respectively.

## All-cause in-hospital mortality

### Patient characteristics

In total, 20/174 (11.5%) patients died during hospitalization, and the all-cause in-hospital mortality was highest in INF2017 (8/36 [22.2%], 9/108 [8.3%] and 3/30 [10.0%] in INF2017, INF2018 and INF2019, respectively). The median time from admission to death was 11.0 (IQR 6.0–17.0) days. Age was higher among influenza patients who died than those who survived; 60.0% (12/20) versus 39.0% (60/154) were aged ≥ 65 years, respectively. Males were more common among those who died as well, 80.0% (16/20) vs. 58.4% (90/154) among patients who survived. The same is true for presence of at least one underlying disease, currently smoking and secondary bacterial pneumonia; 90.0% (18/20) vs. 68.2% (105/154), 50% (10/20) vs. 27.9% (43/154), and 35.0% (7/20) vs. 11.7% (18/154). Administration of neuraminidase inhibitors (including consideration of timing) was not associated with increased mortality risk (p > 0.05) (Table 1).

### Risk factors analysis

**Fig. 1:**
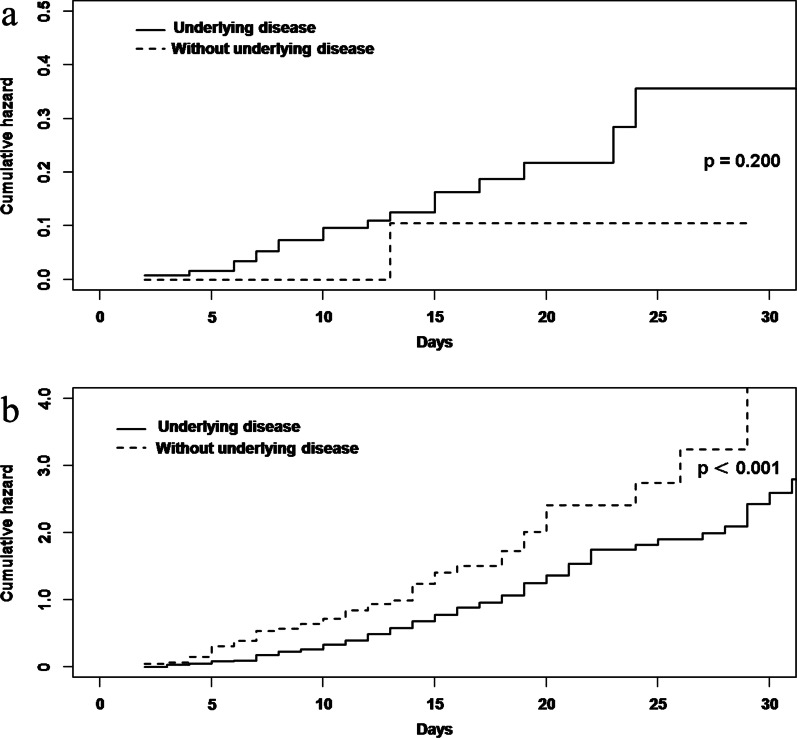
Cumulative hazard curves for **a** all-cause in-hospital mortality and **b** competing risk event (discharge alive) among hospitalized adult patients with community-acquired influenza included in this study, based on the factor, underlying disease

All 174 influenza patients were included in the risk factor analysis. A total of six variables were selected in the univariable analyses: age ≥ 65 years, male gender, current smoker, underlying disease, hypertension and secondary bacterial pneumonia (Additional file 2), and included in the multivariable analysis. The final cause-specific proportional hazards model (AIC = 174.80) for all-cause in-hospital mortality showed time-varying secondary bacterial pneumonia (cause-specific hazard ratio [csHR] 3.38, 95% confidence interval [CI] 1.25–9.17, p = 0.017) was a significant independent risk factor (Table 2). In the cause-specific model for discharge alive, underlying disease (csHR 0.55, 95% CI 0.39–0.77, p = 0.001) decreased the hazard of being discharged alive (increasing length of stay) and as such indirectly increased the risk of mortality (Table 2, Fig. 1).

**Table 2:** Multivariable analysis with cause-specific proportional hazards model for risk factors of all-cause in-hospital mortality among hospitalized adult patients with community-acquired influenza included in this study (n = 174)

Variables	All-cause in-hospital mortality		Competing risk (discharge alive)	
	csHR (95% CI)	p value	csHR (95% CI)	p value
Underlying disease	2.41 (0.56–10.47)	0.239	0.55 (0.39–0.77)	**0.001**
Secondary bacterial pneumonia	3.38 (1.25–9.17)	**0.017**	0.85 (0.51–1.43)	0.541

*csHR* cause-specific hazard ratio

Bold texts indicate p value < 0.05

### Secondary bacterial pneumonia

#### Patient characteristics

Overall, 25/174 (14.4%) patients developed secondary bacterial pneumonia, of which 7 (28.0%) died during hospitalization. The median time from symptom onset to secondary bacterial pneumonia was 8.0 (IQR 4.5–15.0) days. 10/25 (40.0%) of secondary bacterial pneumonia was VAP. Among the patients with secondary bacterial pneumonia, 64.0% (16/25) were aged ≥ 65 years, significantly higher than patients without secondary bacterial pneumonia (37.6% [56/149], p = 0.013); males accounted for 88.0% (22/25) versus 56.4% (84/149) of patients without secondary bacterial pneumonia (p = 0.003). Compared to patients without secondary bacterial pneumonia, a higher proportion of patients with secondary bacterial pneumonia had hypertension (44.0% [11/25] vs. 24.8% [37/149], p = 0.047) and underwent MV (invasive and non-invasive) on admission (44.0% [11/25] vs. 16.1% [24/149], p = 0.001) (Table 1).

#### Risk factors analysis

**Fig. 2:**
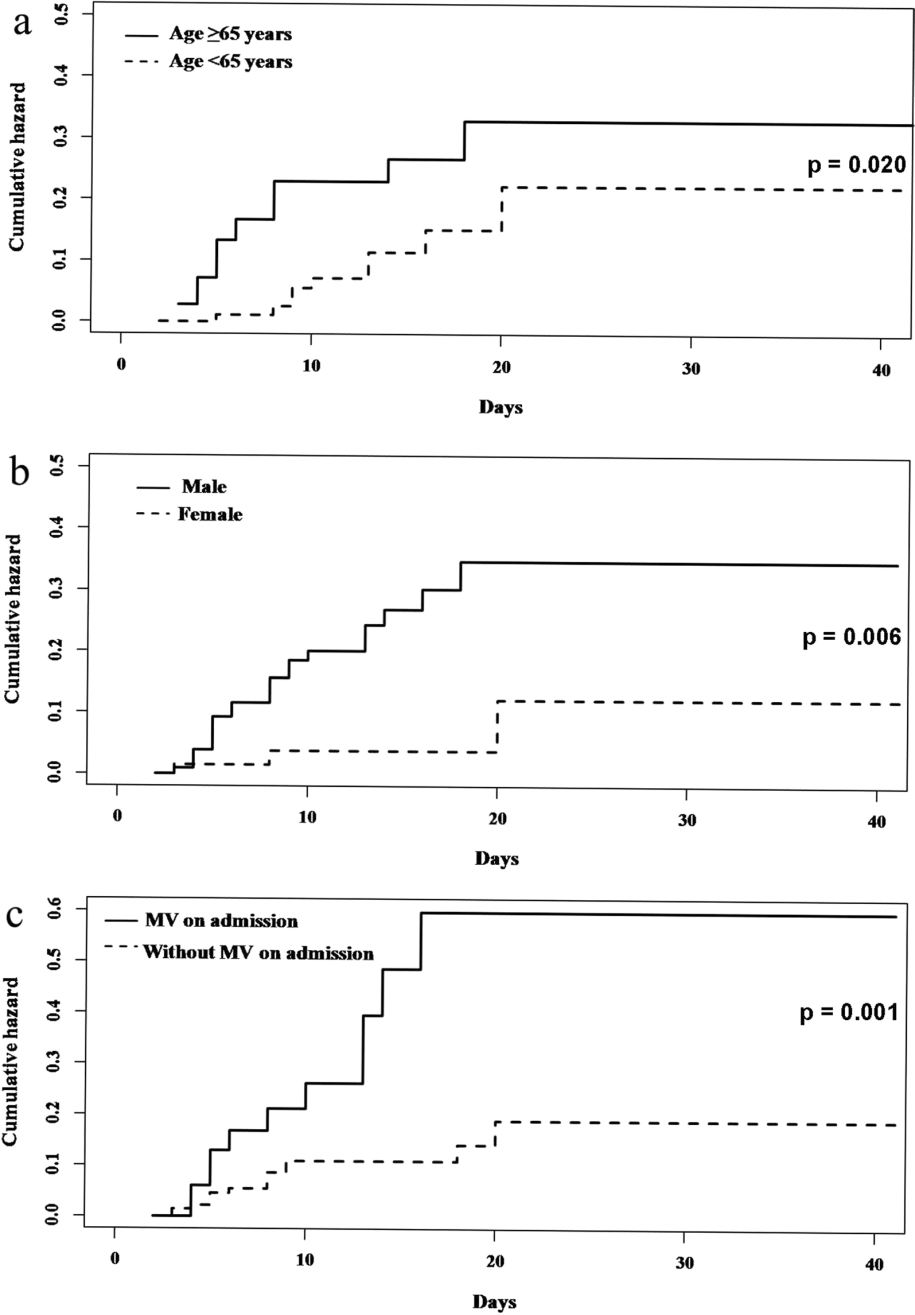
Cumulative hazard curves for secondary bacterial pneumonia among hospitalized adult patients with community-acquired influenza included in this study, based on risk factors: **a** age, **b** gender, and **c** MV on admission. Abbreviation: MV, mechanical ventilation

All 174 influenza patients were included in the risk factor analysis. The multivariable analysis included the following seven variables: age ≥ 65 years, male gender, hypertension, chronic pulmonary disease, anaemia, hypoalbuminemia and MV on admission (Additional file 3). The final cause-specific proportional hazards model (AIC = 219.88) for secondary bacterial pneumonia showed that age ≥ 65 years (csHR 2.90, 95% CI 1.27–6.62, p = 0.012), male gender (csHR 3.78, 95% CI 1.12–12.84, p = 0.033) and MV on admission (csHR 2.96, 95% CI 1.32–6.64, p = 0.008) were independent risk factors for secondary bacterial pneumonia (Table 3, Fig. 2). None of the included variables had an impact on the competing risk of discharge alive or death without secondary bacterial pneumonia (Table 3).

**Table 3:** Multivariable analysis with cause-specific proportional hazards model for risk factors of secondary bacterial pneumonia among hospitalized adult patients with community-acquired influenza included in this study (n = 174)

Variables	Secondary bacterial pneumonia		Competing risk (discharge alive or death without secondary bacterial pneumonia)	
	csHR (95% CI)	p value	csHR (95% CI)	p value
Age ≥ 65 years	2.90 (1.27–6.62)	**0.012**	0.76 (0.54–1.06)	0.105
Male gender	3.78 (1.12–12.84)	**0.033**	0.83 (0.59–1.17)	0.294
MV* on admission	2.96 (1.32–6.64)	**0.008**	0.80 (0.50–1.26)	0.331

*csHR* cause-specific hazard ratio; *MV* mechanical ventilation

Bold texts indicate p value < 0.05

*Invasive and non-invasive MV were included

#### Pathogens of secondary bacterial pneumonia

Pathogens isolated in patients with secondary bacterial pneumonia are shown in Table 4. Among 25 patients with secondary bacterial pneumonia, 35 non-duplicated strains were detected in blood (n = 6), sputum (n = 27) and bronchoalveolar lavage fluid (n = 2) cultures, 9/38 (25.7%) were antimicrobial resistant. The most common pathogens were *Streptococcus pneumoniae*, *Pseudomonas aeruginosa* and *Acinetobacter baumannii* (12/25 [48.0%]). Carbapenem-resistance among the clinical isolates was 100% (4/4) for *Acinetobacter baumannii*, 60% (3/5) for *Pseudomonas aeruginosa*, and 50% (1/2) for *Klebsiella pneumoniae*.

**Table 4:** Pathogens isolated among community-acquired, hospitalized adult influenza patients with secondary bacterial pneumonia included in this study

Pathogen, n (%)	Total patients with secondary bacterial pneumonia (N = 25)	Total non-duplicated* strains (N = 35)	Blood culture (n = 6)	Sputum culture (n = 27)	Bronchoalveolar lavage fluid culture (n = 2)
*Streptococcus pneumoniae*	4 (16.0)	5 (14.3)	1 (16.7)	4 (14.8)	0
*Pseudomonas aeruginosa*	4 (16.0)	5 (14.3)	1 (16.7)	3 (11.1)	1 (50.0)
CRPA	3 (12.0)	3 (8.6)	1 (16.7)	2 (7.4)	0
*Acinetobacter baumannii*	4 (16.0)	4 (11.4)	1 (16.7)	3 (11.1)	0
CRAB	4 (16.0)	4 (11.4)	1 (16.7)	3 (11.1)	0
*Staphylococcus aureus*	3 (12.0)	3 (8.6)	1 (16.7)	2 (7.4)	0
*Haemophilus influenzae*	3 (12.0)	3 (8.6)	0	3 (11.1)	0
*Burkholderia cepacia*	2 (8.0)	3 (8.6)	0	2 (7.4)	1 (50.0)
*Klebsiella pneumoniae*	2 (8.0)	2 (5.7)	0	2 (7.4)	0
CRKP	1 (4.0)	1 (2.9)	0	1 (3.7)	0
*Escherichia coli*	2 (8.0)	2 (5.7)	0	2 (7.4)	0
EPEC	1 (4.0)	1 (2.9)	0	1 (3.7)	0
*Stenotrophomonas maltophilia*	2 (8.0)	2 (5.7)	0	2 (7.4)	0
*Moraxella catarrhalis*	2 (8.0)	2 (5.7)	0	2 (7.4)	0
Others	4 (20.0)	4 (11.4)	2 (33.3)^a^	2 (7.4)^b^	0

*CRPA* Carbapenem-resistant *Pseudomonas aeruginosa*; *CRAB* Carbapenem-resistant *Acinetobacter baumannii*; *CRKP* Carbapenem-resistant *Klebsiella pneumoniae*; *EPEC* Extended spectrum β-lactamase (ESBL)–producing *Escherichia coli*

*If multiple strains of the same bacterial species were obtained from the same specimen source of the same patient, only one strain would be counted

^a^Including *Staphylococcus warneri* (n = 1) and *Staphylococcus epidermidis* (n = 1)

^b^Including *Klebsiella aerogenes* (n = 1) and *Candida albicans* (n = 1)

## Discussion

In this retrospective cohort study, we validated the prior hypothesis that secondary bacterial pneumonia is an important risk factor for all-cause in-hospital mortality among hospitalized adult patients with community-acquired influenza. The effect of secondary bacterial pneumonia on mortality was direct, while underlying disease indirectly increased the risk of mortality through decreasing the hazard of being discharged alive. Then, we identified age ≥ 65 years, male gender and MV on admission as direct risk factors for secondary bacterial pneumonia among hospitalized adult patients with community-acquired influenza.

Our hospital is one of the 554 sentinel hospitals for influenza surveillance in China and has its own laboratory with high quality testing for influenza virus nucleic acid. This greatly avoided ascertainment bias in the study cohort due to low testing rates of influenza virus nucleic acid, frequently reported from other centers. In China, most hospitals need to send samples to the CDC for influenza confirmation, which limits the amount of samples referred.

The all-cause in-hospital mortality of hospitalized adult patients with community-acquired influenza in this study was 11.5%, which is similar to mortality estimates (9.8%) of hospitalized adult patients with influenza over the 2016–2018 influenza seasons at another tertiary hospital in China and that (13.0%) of hospitalized adult patients with influenza from 12 Catalan hospitals in Spain between 2010 and 2016 [23, 24]. A retrospective cohort study in intensive care units of 33 US hospitals over the 2013–2014 showed that the mortality of severely ill patients with influenza was 20.9% [25], revealing that severe cases have a significantly higher mortality. Pneumonia is the most common complication among severe influenza patients, and reported mortality estimates will depend on the attack rate of pneumonia following influenza virus infection. A multicenter cohort study from 2013 to 2019 in China suggested that the 30-day mortality of hospitalized adult patients with influenza-related pneumonia including primary viral pneumonia and secondary bacterial pneumonia was 19.3% [26]. Our study also found a higher in-hospital mortality (28.0%) in influenza patients with secondary bacterial pneumonia, which implies that secondary bacterial pneumonia has a significant impact on survival among influenza patients.

In most previous studies on risk factors for adverse outcomes of patients with influenza, logistic regression model was most frequently statistical method used, while we utilized the cause-specific proportional hazards model which considered the timing of the exposures (secondary bacterial pneumonia) and outcomes (in-hospital mortality and secondary bacterial pneumonia), took into account the impact of competing risks, thus providing a more in-depth understanding of the etiology of a clinical outcome [27, 28]. For in-hospital mortality, discharge alive was considered a competing risk which prevented the observation of the outcome of interest. The cause-specific proportional hazards model showed that secondary bacterial pneumonia didn’t affect discharge alive, but was directly associated with mortality, further confirming the clinical importance of bacterial pneumonia in influenza patients. Neuraminidase inhibitors treatment was not directly associated with mortality in our study, which could be due to the fact that most patients received the treatment rather late. Additionally, it has been shown before that neuraminidase inhibitors treatment for influenza patients can reduce symptom duration, but whether it can reduce the risk of death in hospitalized patients with influenza is still controversial [29].

Influenza virus infection enhances the susceptibility to bacterial infections [30]. A systematic review on clinical outcomes of pandemic influenza A(H1N1)pdm09 infection indicated that secondary bacterial infection was identified in almost one quarter of patients with influenza A(H1N1)pdm09 infection [8]. In this study, the incidence of secondary bacterial pneumonia among hospitalized adult patients with community-acquired influenza was 14.4%, which is slightly higher than the nosocomial infection rate (10.8%) of hospitalized severe influenza A(H1N1)pdm09 patients reported from a study based on multi-center database in China [22]. In our cohort, 20.1% of patients underwent MV on admission, including 13 cases with invasive MV, among which, 46.2% (6/13) developed VAP. And VAP (total 10 cases) accounted for 40.0% of secondary bacterial pneumonia. In line with that, MV on admission was identified as an independent risk factor for secondary bacterial pneumonia, which is confirmed in other studies [6, 22]. Moreover, our study indicated that the impact of MV was direct, not through prolonging length of stay, most likely due to its invasive nature. In light of these results, strict precautions and evidence-based strategies for VAP prevention are essential for influenza patients who are under MV [31]. We also found that age ≥ 65 years and male gender were direct risk factors for secondary bacterial pneumonia. Aging has generally been considered to be associated with infections as immunosenescence results in an impaired immune response [32, 33]. How male gender contributes to secondary bacterial pneumonia is not well understood. Although, a multi-center study in China that found that male gender was a risk factor for influenza A-associated pneumonia severity, hypothesized that a higher frequency of comorbidities and immunosuppression were possible reasons [34].

In this study, the main pathogens of secondary bacterial pneumonia were consistent with those reported in previous studies [5, 6, 8, 9]; *Streptococcus pneumoniae* was one of the most common bacteria. A research report from U.S. CDC showed that *Streptococcus pneumoniae* was the leading bacterial species during the 2009 pandemic influenza A (H1N1), and underscored the importance of pneumococcal vaccination for persons at increased risk for pneumococcal pneumonia [35]. Indeed, only one patient received pneumococcal vaccine in our study. In China, 23-valent pneumococcal polysaccharide vaccine (PPV23) is approved for use in adults to prevent invasive pneumococcal disease. It is reported that the pneumococcal vaccination rate among the elderly in China was only 3.7% in 2019 [36]. The low vaccine coverage may be mainly related to the fact that PPV23, like influenza vaccines, is not part of a national immunization programme, in other words, the cost of vaccination is borne by individuals. The same is true for the influenza vaccine, the average vaccination rate of influenza vaccine in China is also low, 2.43% from 2014 to 2020, with the highest rate 4% in 2020 [37]. Thus, it may be imperative to improve the promotion and related coverage of influenza and pneumococcal vaccine to reduce the important health burden of influenza and secondary bacterial pneumonia.

Our study has several limitations that must be acknowledged. Firstly, our study was retrospective. Several variables that previous studies focused on, such as obesity and immunosuppression, could not be included in this study. Secondly, this was a single-center study with a limited sample size. Specific risk factors for mortality in patients with community-acquired influenza may not have been identified in the final models due to limited statistical power. However, even though the effective sample size for mortality risk factor analysis was indeed small, the prior hypothesis that secondary bacterial pneumonia was an important risk factor could still be verified. In addition, our hospital is one of the largest tertiary teaching hospitals in southwest China.

## Conclusions

Secondary bacterial pneumonia was a major direct risk factor for all-cause in-hospital mortality among hospitalized adult patients with community-acquired influenza in a large Chinese tertiary hospital. Additionally, underlying disease indirectly increased death risk through prolonging length of stay. Among influenza patients, prevention of secondary bacterial pneumonia is paramount, and clinicians and epidemiologists need to pay particular attention to prevention measures for elderly, male patients, and strengthen the monitoring of and prevention measures against VAP for patients under MV. To reduce the public health burden of influenza, efforts including vaccination promotion must be strengthened to reduce the attack rate of influenza virus and secondary *Streptococcus pneumoniae* infection and improve public health outcomes.

## Supplementary Information

**Additional file 1**: Collected variables for risk factor analysis of secondary bacterial pneumonia and in-hospital mortality among hospitalized adult patients with community-acquired influenza.

**Additional file 2**: Univariable analysis with Cox regression for risk factors of all-cause in-hospital mortality among hospitalized adult patients with community-acquired influenza included in this study.

**Additional file 3**: Univariable analysis with Cox regression for risk factors of secondary bacterial pneumonia among hospitalized adult patients with community-acquired influenza included in this study.

## Data Availability

The datasets supporting the conclusions of this article are included within the article (Tables 1, 2, 3, and 4) and Additional files 1, 2, and 3.

## Abbreviations

AIC: Akaike’s Information Criteria
CDC: Centers for Disease Control and Prevention
COPD: Chronic obstructive pulmonary disease
CRAB: Carbapenem-resistant *Acinetobacter baumannii*
CRKP: Carbapenem-resistant *Klebsiella pneumoniae*
CRPA: Carbapenem-resistant *Pseudomonas aeruginosa*
csHR: Cause-specific hazard ratio
EPEC: Extended spectrum β-lactamase (ESBL)–producing *Escherichia coli*
IQR: Interquartile range
MV: Mechanical ventilation
PH: Proportional hazard
PPV23: 23-Valent pneumococcal polysaccharide vaccine
RT-PCR: Real-time reverse transcriptase polymerase chain reaction
TB: Tuberculosis
VAP: Ventilator-associated pneumonia
