# Supplementary material for: Risk factors for in-hospital mortality and secondary bacterial pneumonia among hospitalized adult patients with community-acquired influenza: a large retrospective cohort study
Source: Antimicrob Resist Infect Control. 2023 Mar 31;12:25. doi: 10.1186/s13756-023-01234-y (PMC10064953; doi:10.1186/s13756-023-01234-y)
Supplement: Supplementary file 1 — Additional file 1: Collected variables for risk factor analysis of secondary bacterial pneumonia and in-hospital mortality among hospitalized adult patients with community-acquired influenza. [file 13756_2023_1234_MOESM1_ESM.docx]

**Additional file 1. Collected variables for risk factor analysis of secondary bacterial pneumonia and in-hospital mortality among hospitalized adult patients with community-acquired influenza**

| **Category** | **Variables** |
| --- | --- |
| Demographics | age, gender |
| Epidemiology | influenza season, type of influenza virus, date of symptom onset, current smoker, influenza vaccine >14 days before symptom onset in the same season |
| Hospitalization information | admission date, discharge date |
| Underlying disease | hypertension, diabetes, chronic pulmonary disease, chronic heart disease, chronic renal disease, chronic liver disease, haematological disease, cerebrovascular disease, malignancy |
| Self-reported symptoms on admission | fever, cough, sputum, sore throat, haemoptysis, dyspnoea, chest pain, muscle or body aches, headaches, fatigue, gastrointestinal symptoms |
| The laboratory findings on admission | neutrophil count, lymphocyte count, platelet count, haemoglobin, albumin |
| The first radiographic findings after admission | pleural effusion, diffuse bilateral pulmonary infiltration |
| Treatments | mechanical ventilation (invasive and non-invasive) on admission, neuraminidase inhibitors treatment, start date of neuraminidase inhibitors treatment, duration of neuraminidase inhibitors treatment |
| Secondary bacterial pneumonia | secondary bacterial pneumonia, date of secondary bacterial pneumonia, microbiological characteristics of causative pathogen(s) (pathogen, specimen source and antimicrobial resistance) |
| All-cause in-hospital death | death, date of death |
