# Supplementary material for: Risk factors for in-hospital mortality and secondary bacterial pneumonia among hospitalized adult patients with community-acquired influenza: a large retrospective cohort study
Source: Antimicrob Resist Infect Control. 2023 Mar 31;12:25. doi: 10.1186/s13756-023-01234-y (PMC10064953; doi:10.1186/s13756-023-01234-y)
Supplement: Supplementary file 2 — Additional file 2: Univariable analysis with Cox regression for risk factors of all-cause in-hospital mortality among hospitalized adult patients with community-acquired influenza included in this study. [file 13756_2023_1234_MOESM2_ESM.docx]

**Additional file 2. Univariable analysis with Cox regression for risk factors of all-cause in-hospital mortality among hospitalized adult patients with community-acquired influenza included in this study**

| **Variables** | **HR (95% CI)** | **p value** |
| --- | --- | --- |
| **Age ≥65 years** | 1.84 (0.75-4.50) | 0.181 |
| **Male gender** | 2.11 (0.71-6.30) | 0.181 |
| **Underlying disease** | 2.58 (0.60-11.15) | 0.194 |
| Hypertension | 1.96 (0.81-4.80) | 0.135 |
| Diabetes | 1.14 (0.43-3.00) | 0.789 |
| Chronic pulmonary disease | 0.54 (0.12-2.30) | 0.406 |
| COPD | 0.60 (0.14-2.60) | 0.488 |
| Asthma | 3.20 (0.42-24.00) | 0.259 |
| TB | - | 0.997 |
| Chronic heart disease^a^ | 1.28 (0.47-3.50) | 0.628 |
| Chronic renal disease | 1.05 (0.24-4.60) | 0.953 |
| Chronic liver disease | 1.06 (0.35-3.20) | 0.919 |
| Haematological disease | 0.52 (0.07-3.90) | 0.523 |
| Cerebrovascular disease | - | 0.997 |
| Malignancy | 0.85 (0.20-3.70) | 0.824 |
| **Current smoker** | 1.91 (0.79-4.60) | 0.150 |
| **Neuraminidase inhibitors treatment** | 1.02 (0.14-7.70) | 0.984 |
| Neuraminidase inhibitors treatment ≤48h since symptom onset | 0.99 (0.36-2.70) | 0.991 |
| **Secondary bacterial pneumonia** | 3.53 (1.30-9.56) | 0.013 |

HR: hazard ratio; CI: confidence interval; COPD: chronic obstructive pulmonary disease; TB: tuberculosis.

^a^ Chronic heart disease included coronary heart disease, congestive heart failure, rheumatic heart disease, hypertensive heart disease, cor pulmonale and congenital heart disease.
