# Supplementary material for: Risk factors for in-hospital mortality and secondary bacterial pneumonia among hospitalized adult patients with community-acquired influenza: a large retrospective cohort study
Source: Antimicrob Resist Infect Control. 2023 Mar 31;12:25. doi: 10.1186/s13756-023-01234-y (PMC10064953; doi:10.1186/s13756-023-01234-y)
Supplement: Supplementary file 3 — Additional file 3: Univariable analysis with Cox regression for risk factors of secondary bacterial pneumonia among hospitalized adult patients with community-acquired influenza included in this study. [file 13756_2023_1234_MOESM3_ESM.docx]

**Additional file 3. Univariable analysis with Cox regression for risk factors of secondary bacterial pneumonia among hospitalized adult patients with community-acquired influenza included in this study**

| **Variables** | **HR (95% CI)** | **p value** |
| --- | --- | --- |
| **Age ≥65 years** | 2.56 (1.10-5.80) | 0.024 |
| **Male gender** | 4.69 (1.40-16.00) | 0.012 |
| **Underlying disease** | 1.76 (0.60-5.20) | 0.299 |
| Hypertension | 2.08 (0.95-4.60) | 0.069 |
| Diabetes | 1.50 (0.65-3.50) | 0.344 |
| Chronic pulmonary disease | 1.91 (0.80-4.60) | 0.146 |
| COPD | 1.73 (0.69-4.30) | 0.242 |
| Asthma | 2.39 (0.32-18.00) | 0.394 |
| TB | 1.91 (0.26-14.00) | 0.526 |
| Chronic heart disease^a^ | 0.71 (0.24-2.10) | 0.535 |
| Chronic renal disease | 0.45 (0.06-3.30) | 0.434 |
| Chronic liver disease | 0.57 (0.17-1.90) | 0.362 |
| Haematological disease | 0.43 (0.06-3.20) | 0.412 |
| Cerebrovascular disease | 1.43 (0.34-6.10) | 0.629 |
| Malignancy | 1.39 (0.48-4.10) | 0.546 |
| **Current smoker** | 1.63 (0.74-3.60) | 0.229 |
| **The laboratory findings on admission** |  |  |
| Neutropenia^b^ | - | 0.997 |
| Lymphocytopenia^c^ | 1.85 (0.69-5.00) | 0.224 |
| Thrombocytopenia^d^ | 1.07 (0.43-2.70) | 0.887 |
| Anaemia^e^ | 2.28 (1.00-5.00) | 0.039 |
| Hypoalbuminemia^f^ | 3.05 (1.40-6.70) | 0.005 |
| **Mechanical ventilation^g^ on admission** | 3.35 (1.50-7.40) | 0.003 |

HR: hazard ratio; CI: confidence interval; COPD: chronic obstructive pulmonary disease; TB: tuberculosis.

^a^ Chronic heart disease included coronary heart disease, congestive heart failure, rheumatic heart disease, hypertensive heart disease, cor pulmonale and congenital heart disease.

^b^ Neutropenia: neutrophil count <1,500/mm^3^.

^c^ Lymphocytopenia: lymphocyte count <800/mm^3^.

^d^ Thrombocytopenia: platelet count <100,000/mm^3^.

^e^ Anaemia: haemoglobin <120 g/L for men and <110 g/L for women.

^f^ Hypoalbuminemia: albumin <35 g/L.

^g^ Invasive and non-invasive mechanical ventilation were included.
